# Efficacy and Safety of Bariatric Surgery in Dutch People Living with HIV: a Retrospective Matched Cohort Analysis

**DOI:** 10.1007/s11695-024-07126-3

**Published:** 2024-03-04

**Authors:** Leena Zino, Rou Qing Chen, Laura Deden, Eric Hazebroek, Olivier Richel, Angela Colbers, David M Burger, On behalf of the DATO Research Group

**Affiliations:** 1 Department of Pharmacy and Radboudumc Research Institute for Medical Innovation (RIMI), Radboud University Medical Center, 864 Radboudumc, Geert Grooteplein 10, 6525 GA Nijmegen, The Netherlands; 2 Department of Bariatric Surgery, Vitalys Clinic, Rijnstate Hospital, Arnhem, The Netherlands; 3 Department of Internal Medicine and Radboudumc, Division Infectious Diseases, Radboud University Medical Center, Nijmegen, The Netherlands

**Keywords:** HIV, Bariatric surgery, Controls, TWL, Safety

## Abstract

**Purpose:**

Obesity is rising among people with HIV (PLWH), sparking interest in bariatric surgery (BS) for this group. Yet, large-scale comparative research on BS outcomes in PLWH is lacking.

**Methods:**

We performed a retrospective, matched cohort analysis in PLWH and HIV uninfected controls. Subjects were retrieved from the Dutch Audit for Treatment of Obesity (DATO) registry. Matching (1:7 ratio) included age (± 5-years), sex, body-mass index (BMI) of ± 3 kg/m^2^, surgery type, and associated health problems (AHPs) at baseline. The primary endpoint was total weight loss percentage (%TWL) ≥ 20% achieved at 1-year post-BS. Secondary endpoints were cumulative %TWL achieved at 2-years post-BS, a reported remission or improvement in AHPs post-BS, and surgical complications, both at 1-year post-BS. Comparisons were performed using conditional logistic regression.

**Results:**

Twenty-seven PLWH and 168 controls were included. At 1-year post-BS, 89% PLWH achieved ≥ 20%TWL, compared to 94% of controls (*p* = 0.4). Cumulative %TWL at 2-years post-BS were 82% and 92% in PLWH and controls, respectively (*p* = 0.2). Improvement rates in hypertension and type 2 diabetes mellitus were 50% and 86% in PLWH, versus 87% and 87% in controls. Full remission occurred in 20% and 71% of PLHIV, versus 49% and 44% of controls, respectively. No improvement or remission was observed for dyslipidaemia in PLHIV compared to 54% improvement and 29% remission in controls. Surgical complications were 0% in PLHIV and 13% (*n* = 21) in controls.

**Conclusion:**

Efficacy and safety outcomes of BS were similar between PLWH and controls except for the lack of improvement in dyslipidaemia in PLWH.

**Graphical abstract:**

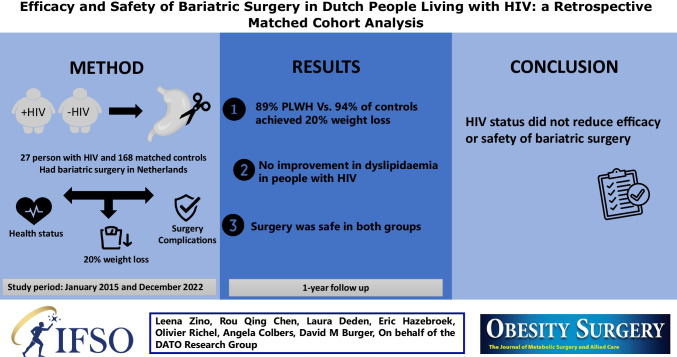

**Supplementary Information:**

The online version contains supplementary material available at 10.1007/s11695-024-07126-3.

## Introduction

Overweight and obesity trends are increasing across all populations, including people living with HIV (PLWH). Nowadays, the prevalence of obesity in men and women living with HIV is about 19% and 49%, respectively [1]. The high prevalence of obesity in PLWH is parallel to those in the general population, because of a shared obesogenic environment [2]. Additionally, advances in combination antiretroviral therapy (cART) have significantly improved survival in PLWH allowing for similar aging-driven health problems, such as obesity [1, 3]. Moreover, cART initiation is likely to contribute to weight gain in PLWH [1, 4]. Several mechanisms of weight gain after cART initiation have been proposed, including a reversal of the accelerated catabolism characteristic of an active HIV infection, and resolution of gastrointestinal dysfunction that could adversely affect appetite and nutrient absorption [5].

Not limited to obesity, HIV is an independent risk factor for cardiovascular disease (CVD) and metabolic syndromes among other associated health problems (AHPs) [6, 7], probably due to the immune dysregulation and cART toxicity [2, 8]. Reports show higher rates of CVD, type 2 diabetes mellitus (T2DM), hypertension and dyslipidaemia in PLWH on cART compared to uninfected individuals with similar demographics [6, 7, 9–11]. As those AHPs are major features of metabolic syndromes and core drivers of cardiovascular risk, it is of great importance to tackle obesity in PLWH.

Bariatric surgery (BS) is an effective and sustainable method for weight loss in the general population when diet and lifestyle changes fail to achieve weight goals [12]. Besides weight loss, BS can also improve AHPs of obesity, such as hypertension, dyslipidaemia and T2DM in the general population [13]. Similar results in PLWH are yet to be fully investigated. Sharma et al. conducted a retrospective, case–control study in 11 PLWH (+ 55 controls) and suggested no significant difference in short-term surgical complications or metabolic outcomes between the groups [14]. Although this study suggested similar BS outcomes between PLWH and the non-infected population, it was limited by the small sample size, single-centre nature, and lack of standardization in surgery technique by the time of analysis. Thus, large-scale, comparative research in PLWH is lacking which results in a paucity of clinical guidelines on the efficacy and safety of performing BS in PLWH.

The aim of this study was to conduct a matched-cohort analysis to investigate the impact of an HIV status on the efficacy and safety outcomes of BS in the Netherlands.

## Methods

### Study Cohort

Adult patients (≥ 18 years) who underwent a primary sleeve gastrectomy (SG) or Roux-en-Y gastric bypass (RYGB) procedure in the Netherlands between January 2015 and December 2022 were retrospectively identified for this analysis from the Dutch Audit for Treatment of Obesity (DATO). DATO is a prospective, mandatory national registry for all BS clinics in the Netherlands since 2015. All registered patients underwent a surgical intervention due to severe obesity (BMI ≥ 40 kg/m^2^), or a BMI ≥ 35 kg/m^2^ accompanied with ≥ 1 major AHPs of obesity (hypertension, T2DM, dyslipidaemia, obstructive sleep apnoea syndrome (OSAS), gastroesophageal reflux disease (GERD) or musculoskeletal pain) [15, 16]. No informed consent was needed as this is an opt-out registry and the study has been performed in accordance with the ethical standards as stated in Dutch law and the regulations of the Dutch Institute for Clinical Auditing (DICA). Subjects with or without HIV were matched in a 1:7 ratio using DATO independent input on HIV status. Matching included age (± 5 years), sex, surgical type, baseline BMI (± 3 kg/m^2^), and baseline status of hypertension, T2DM and dyslipidaemia.

### Data Collection and Definitions

Data collected at baseline included demographics, surgery type and date, and relevant AHPs (cerebrovascular disease, liver disease, kidney disease, HIV/acquired immune deficiency syndrome (AIDS), hypertension, T2DM, dyslipidaemia, OSAS, GERD and musculoskeletal pain). Pre-operative status of AHPs were categorized as: absent, present, present with medication. A full description of the presence of AHPs at baseline is depicted in Table S1 (Supplementary material). Post-operatively, follow-up data were yearly assessed and included weight, status of AHPs of obesity, and post-surgical complications. Improvement and full remission of hypertension, T2DM and dyslipidaemia were defined following the American Society for Metabolic and Bariatric Surgery [17], and summarized in Table S2 in the Supplementary. Surgical complications were categorized into short-term (≤ 30 days), or long-term (> 30 days), as well as minor or major complications according to Clavien-Dindo classification [18]. Also, days of hospital stay post-surgery, and readmission or intensive care unit (ICU) administration for a surgical complication were assessed.

### Primary and Secondary Endpoints

The primary endpoint was to compare weight reduction between PLWH and the uninfected controls at 1 year post-BS. Achieving a percentage of total weight loss (%TWL) ≥ 20% at 1-year post-BS is currently recommended to measure the weight loss efficacy post-BS and, therefore, was used for this analysis [17, 19]. The %TWL was calculated using the following formula:

$$\frac{Pre\;operative\;weight\;-\;Post\;operative\;weight}{Pre\;operative\;weight}*100\mathrm{\% }\left[19\right].$$

Secondary endpoints were the cumulative %TWL at 2-years post-BS, the incidence of improvement or full remission of hypertension, T2DM and dyslipidaemia, and the incidence of short- and long-term surgical complications represented by the overall number of complications, grade of complication, the need for hospital readmission or ICU admission for these complications, all at 1-year post-BS.

### Statistical Analysis

Descriptive statistics were used to summarize the baseline characteristics of the patients. Normally distributed, continuous variables were displayed as a mean with standard deviation (SD), while non-normal distributed variables were displayed as the median with interquartile range (IQR). Categorical variables were displayed as counts and percentages and were compared using descriptive statistics. Independent samples t-test was used to compare continuous variables. Conditional logistic regression analysis was used to determine the association between an HIV infection and %TWL above or below 20%, displayed an odds ratio (OR) with 95% confidence interval (CI). Significance was set at *p* < 0.05. Data analysis and visualization were done using SPSS v27 (IBM Corp., Armonk, NY, USA) and GraphPad Prism v9.

## Results

### Subject Characteristics

**Fig. 1:**
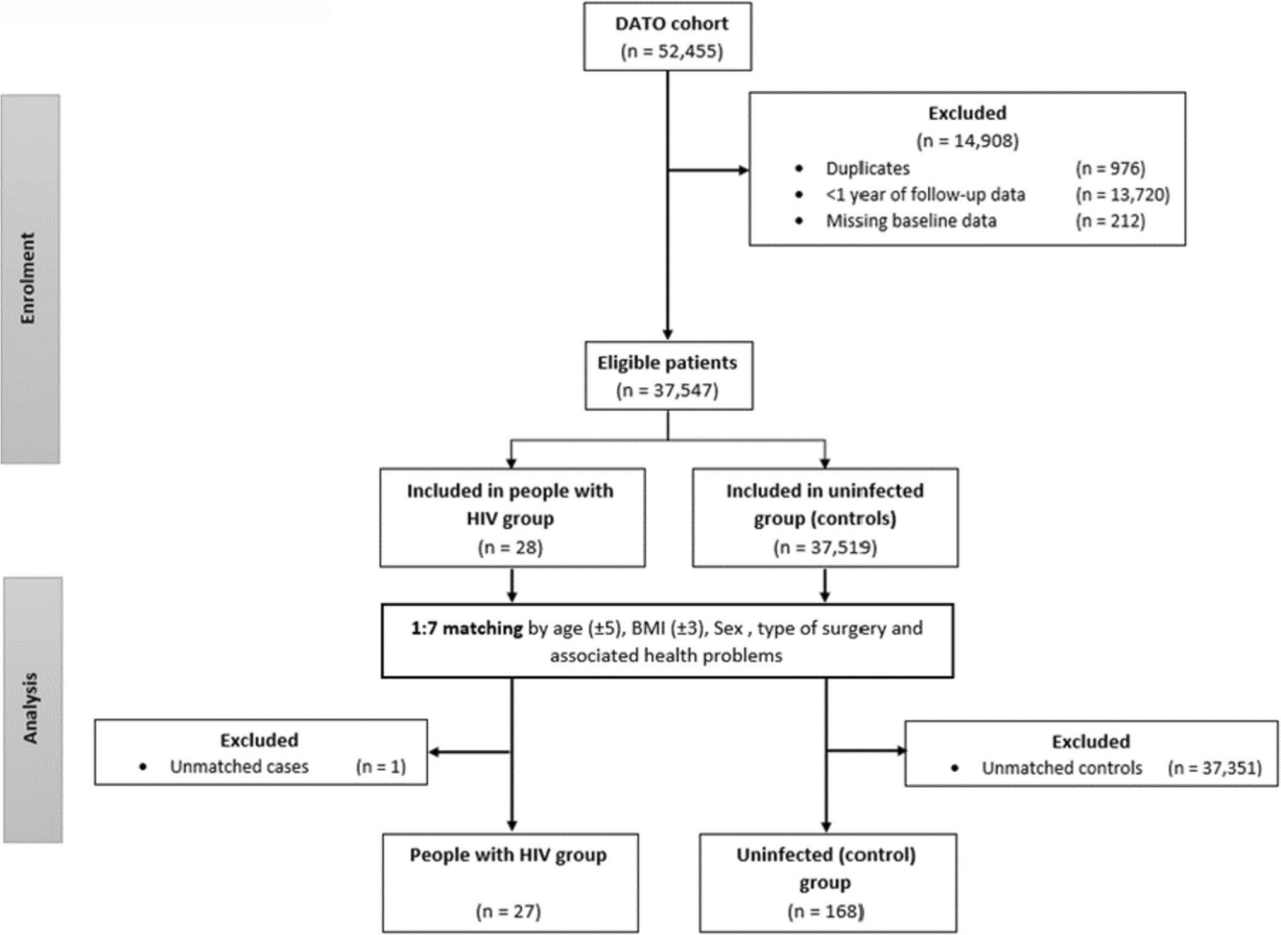
Flow chart showing the processing of the original dataset into the population of this study

52,455 adult patients who underwent an RYGB or SG procedure in the Netherlands between January 2015 and December 2022 were registered in DATO dataset. After removing duplicates, patients with missing baseline or 1-year follow up data, 37,547 patients remained. Twenty-eight subjects had an HIV indication confirmed by a PCR test. One subject with HIV was excluded because there were no available matched controls due to a relatively young age (18-years) and extremely high BMI (> 60 kg/m^2^). Maintaining the ratio of 1:7 matched controls was not possible for 4 individuals with HIV, therefore, fewer matches (1, 1, 2, and 3 matches) were found and added to the dataset. A total of 27 PLWH and 168 matched controls were included. Inclusion strategy is shown in Fig. 1. Median age was 49 years for both populations. None of the matched baseline variables differed between the study groups (*p* = 0.8 to 0.99). Among the unmatched AHPs, a greater proportion of the uninfected controls had musculoskeletal pain at baseline. Full cohort characteristics are depicted in Table 1.

**Table 1:** Characteristics of the study cohorts

Variable	People living with HIV (*N* = 27)	Uninfected controls (*N* = 168)
Median age at surgery, years (IQR)	49 (42–55)	49 (44–54)
Sex, n (%)		
Male	11 (40.7)	66 (39.3)
Female	16 (59.3)	102 (60.7)
Median preoperative weight, kg (IQR)	117.6 (103.1–140.0)	122.1 (112.2–140.2)
Median preoperative BMI, kg/m^2^ (IQR)	41.33 (37.99–47.33)	41.26 (39.05–46.48)
Type of surgery, n (%)		
SG	13 (48.1)	76 (45.2)
RYGB	14 (51.9)	92 (54.8)
Preoperative AHPs, n (%)		
Hypertension	10 (37.0)	53 (31.6)
T2DM	7 (25.9)	45 (26.8)
Dyslipidaemia	9 (33.3)	48 (28.6)
Additional unmatched AHPs, n (%)		
GERD	4 (14.8)	28 (16.7)
OSAS	7 (25.9)	47 (25.0)
Musculoskeletal pain	7 (25.9)	83 (49.4)
Cerebrovascular disease	1 (3.7)	7 (4.2)
Liver disease	1 (3.7)	0 (0.0)
Kidney disease	1 (3.7)	1 (0.6)

*AHPs* associated health problems

### Weight Loss Post-BS

Eighty-nine percent (*n* = 24) of PLWH achieved a TWL ≥ 20% at 1-year post-BS compared to 94% (*n* = 158) of controls. At 2-years post-BS, these percentages decreased to 81.5% and 91.7% for PLWH and controls, respectively. Incidence of achieving a TWL ≥ 20% was not statistically different between people with or without HIV at 1- and 2-year post-surgery. Mean percentage TWL (SD) was comparable between the two groups at 1-year post-BS (29.9% (10.9) in PLWH and 29.8% (6.7) in controls), while it was slightly lower in PLWH at 2-years post-BS (27.6% (12.9)) than in controls (30% (8.5)). Mean differences in percentage of TWL were not statistically significant between the two groups at 1 or 2-years post-BS, even when patients were stratified by surgery type (Figures S1-S2, supplementary material, *p* value = 0.2) (Tables 2 and 3).

**Table 2:** Total weight loss at 1 and 2-years post-surgery in people with and without HIV

Cohort	1-year post-surgery		2-years post-surgery	
	≥ 20% TWL, *n* (%)	OR (95% CI)*	≥ 20% TWL, *n* (%)	OR (95% CI)*
People living with HIV	24 (89%)	0.5 (0.13–2.0)	22 (81.5%)	0.5 (0.2–1.4)
Uninfected Controls	158 (94%)	Reference	154 (91.7%)	Reference

*Conditional logistic regression analysis

**Table 3:** Improvement and remission of associated health problems at 1-year post-BS

	People living with HIV (*N* = 27)	Uninfected controls (*N* = 168)
Improvement of associated health problems, *n*/*N* (%)		
Hypertension	5/10 (50.0)	46/53 (86.8)
Diabetes mellitus type 2	6/7 (85.7)	39/45 (86.7)
Dyslipidaemia	0/9 (0.0)	26/48 (54.2)
Remission of associated health problems, *n*/*N* (%)		
Hypertension	2/10 (20.0)	26/53 (49.1)
Diabetes mellitus type 2	5/7 (71.4)	20/45 (44.4)
Dyslipidaemia	0/9 (0.0)	14/48 (29.2)

### Improvement in AHPs of Obesity

After one year, 5 out of 10 PLWH (50%) had an improvement of their hypertension compared to 46/53 (86.8%) in controls. Hypertension remission occurred in 2 subjects with HIV (20%) versus 26 (49.1%) in controls at the end of the year. Improvement of T2DM was similarly high in PLWH (85.7%) and controls (86.7%). Interestingly, rates of T2DM remission at 1-year post-BS were slightly higher in PLWH (5/7, 71.4%) compared to 20/45 (44.4%) in uninfected controls, although numbers of PLWH were low. There was no improvement or remission in dyslipidaemia among PLWH at 1-year post-BS, while 26 (54.2%) uninfected controls had an improvement in dyslipidaemia, and 14 (29.2%) had a full remission. Due to the low number of events per AHP in PLWH (*n* < 10), descriptive statistics were used to describe improvement and remission rather than conditional logistic regression.

### Surgical Complications

There were no reported surgical complications in PLWH at 1-years post-BS. However, in the uninfected controls, 21 subjects (12.5%) presented with at least one surgery complication. Of these, 11 subjects presented short-term, and 10 presented long-term complications. No grade IV or V events were reported (Clavien Dindo Classification). Table S3 summarizes all reported surgical complications in this analysis.

## Discussion

This study aimed to evaluate the efficacy and safety outcomes of BS in Dutch PLWH compared to uninfected matched controls. The presence of HIV did not result in a statistically significant difference in short-term weight loss post-BS nor increased the risk of surgery complications.

A large majority in both study groups achieved ≥ 20% TWL at 1-year post-BS in our analysis. Mean %TWL (SD) at 1-year post-BS was comparable across groups at around 29.9% (10.90) versus 29.8% (6.67). These values of %TWL were similar in other studies conducted in the general population showing a mean %TWL at 1-year follow-up between 24.2–37.1% after RYGB, and between 26.3–34.8% after SG [20]. At 2-years post-BS, %TWL in PLWH was not statistically different from that of the uninfected controls. This observation is important because multiple first line cART options as well as low CD4^+^ T-cell count during HIV infection are associated with weight gain in PLWH [14]. Nevertheless, our analysis suggested that these risk factors do not significantly influence the weight loss outcomes in PLWH up to 2-years of follow-up.

The improvement and remission rates of two major obesity AHPs, hypertension and T2DM, but not dyslipidaemia, were comparable between the two cohorts in this analysis. A study published in 2018 showed similar differences in remission of AHPs between 11 PLWH and their matched uninfected controls. Yet, higher rates of T2DM remission were observed in PLWH in our analysis which is unlikely to be clinically significant and could be attributed mainly to the smaller sample size for the PLWH cohort.

The same study also reported comparable rates of dyslipidaemia remission at 1-year post-BS [14] which we did not detect in our PLWH cohort. This could be attributed to the lipid disruption effect of some newer cART that were available for PLWH during the period of our analysis. A multivariate analysis on a large cohort (*n* = 2343) reported that starting cART containing the newer antiretrovirals, elvitegravir and tenofovir alafenamide fumarate, was associated with a significantly higher risk of hypercholesterolemia [adjusted hazard ratio (HR) of 4.12], hypertriglyceridemia (adjusted HR, 1.69), and high LDL-C (adjusted HR of 4.60) compared to those who started older agents, efavirenz and tenofovir disoproxil fumarate [21]. Although the effect of elvitegravir and tenofovir alafenamide fumarate on the lipid profile has been confirmed by another study [22], a wide generalization of this observation to all newer cART options is not evidenced.

We did not find any documented surgical complication in PLWH, while 22/168 non-infected patients had a surgical complication. Similarly, McCarty et al. showed similar in-hospital mortality and of postprocedural complications between patients with and without HIV [23]. Another study demonstrated similar results regarding post-operative complications [14]. Although our results are in consistence with the literature, a reporting bias is plausible as PLWH may report some complications to their HIV physician rather than the bariatric surgeon and thus, will not be reported in our database.

Our study has few limitations. Matching was done on major cofounders known to alter BS outcomes, thus limiting confounding bias. Yet, there is a possibility of a residual confounding effect that we could not cover via our matching, as some covariates i.e., CD4^+^ T-cell count and type of HIV treatment, were not recorded in the dataset. The lack of data on HIV treatment may limit the interpretation of how these treatments contribute to higher incidence of AHPs such as dyslipidaemia or T2DM. Besides, information such as weight management methods, lifestyle modification and pharmacotherapies, which could influence weight loss outcome post-BS, were not available. Additionally, the selection of patients who had the surgery in 2018 onwards did not allow for a longer follow-up in this analysis. However, this was chosen because since 2018 the BS procedure has been fully standardized in the Netherlands, which reduced the effect of surgery technique on the final outcomes. Another limitation for this study was the small sample size which particularly restricts the statistical comparison of AHPs remission or improvement between HIV positive and negative populations. Nevertheless, people living with HIV who undergo bariatric surgery is considered a small special population, with this study being the biggest comparative study in the literature. Furthermore, this analysis included prospectively registered data from all eighteen bariatric centres in the Netherlands. This minimizes the possible bias of single-centre studies and allows better generalizability of Dutch clinical practices.

In conclusion, our matched-cohort analysis demonstrated that BS was associated with similar short-term weight loss among people with or without HIV and no increased risk of surgical complications. Improvement and remission of AHPs of obesity at 1-year post-BS was comparable between both groups except for dyslipidaemia in PLWH where no improvement or remission was documented up to 1-year post-BS. These findings indicate that BS does not present a greater risk of reduced efficacy or compromised safety in people with HIV compared to matched individuals without HIV.

### Supplementary Information

Below is the link to the electronic supplementary material.

Supplementary file1 (DOCX 57 KB)

## Data Availability

Data are available upon request from DATO research department.
