# Supplementary material for: Efficacy and Safety of Bariatric Surgery in Dutch People Living with HIV: a Retrospective Matched Cohort Analysis
Source: Obes Surg. 2024 Mar 4;34(5):1584–9. doi: 10.1007/s11695-024-07126-3 (PMC11031456; doi:10.1007/s11695-024-07126-3)
Supplement: Supplementary file 1 — Supplementary file1 (DOCX 57 KB) [file 11695_2024_7126_MOESM1_ESM.docx]

# Supplementary material

Table S1 Definition of associated health problems of obesity at baseline summarized from [1]

|  | **Definition** |
| --- | --- |
| **Hypertension** | Present if: pre-hypertension (SBP 120-140 mmHg, DBP 80-90 mmHg), hypertension stadium I (SBP 140-160 mmHg, DBP 90-100 mmHg), and hypertension stadium II (SBP >160 mmHg, DBP >100 mmHg). |
| **Dyslipidaemia** | Present if: divergent lipid spectrum (LDL-c >2.6 mmol/L, HDL-c <1.03 mmol/L, and triglycerides >1.7 mmol/L). |
| **OSAS** | Present if: symptoms, positive PSG, possibly in combination with an AHI of >5. |
| **GERD** | Present if: anamnestic indication of GERD, possibly in combination with a positive 24–48-hour pH measurement and/or gastroduodenoscopy. |
| **Musculoskeletal pain** | Present if: there is a current diagnosis of (lumbos)sacral arthrosis, coxarthrosis, gonarthrosis, osteoarthritis and/or (other) musculoskeletal pain for which specialist treatment is indicated. |
| *SBP* systolic blood pressure; *DBP* diastolic blood pressure; *LDL-c* low-density lipoprotein cholesterol; *HDL-c* high-density lipoprotein cholesterol; *OSAS* obstructive sleep apnoea syndrome; *PSG* polysomnography; *AHI* apnoea-hypopnoea index; *GERD* gastroesophageal reflux disease | |

Table S2 Associated health problems of obesity and their definition of improvement and remission summarized from [1]

|  | **Status** | **Definition** |
| --- | --- | --- |
| **Hypertension** | Improvement | Decrease in dosage and/or number of antihypertensive medications or decrease in SBP and/or DSB on the same medication. |
|  | Remission | Normotensive blood pressure values (<120/80 mmHg) off antihypertensive medications. |
| **T2DM** | Improvement | Statistically significant reduction in HbA1c (≤69 mmol/mol) and FBG not meeting criteria for remission and/or decrease in antidiabetic medications requirement (by discontinuing insulin or one oral agent, or 50% reduction in dose). |
|  | Remission | Normal measures of glucose metabolism (HbA1c <53 mmol/mol) off antidiabetic medications. |
| **Dyslipidaemia** | Improvement | Decrease in dosage and/or number of lipid-lowering medication with equivalent control of dyslipidaemia or improved control of lipids on equivalent medication. |
|  | Remission | Normal lipid panel (LDL-c <2.6 mmol/L, HDL-c >1.03 mmol/L and triglyceride <1.7 mmol/L) off lipid-lowering medication. |
| *SBP* systolic blood pressure; *DBP* diastolic blood pressure; *HbA1c* glycated haemoglobin type 1Ac; *FBG* fasting blood glucose; *LDL-c* low-density lipoprotein cholesterol; *HDL-c* high-density lipoprotein cholesterol | | |


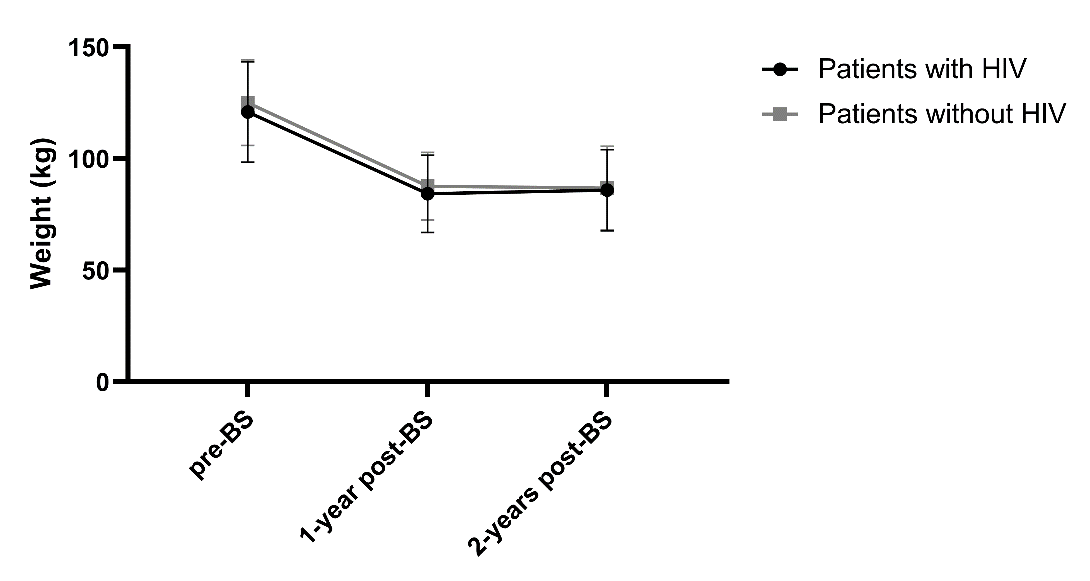


Figure S1. Mean weight in kg (SD) of people with and without HIV up to 2-years after bariatric surgery (BS) at 1 and 2-years post-BS


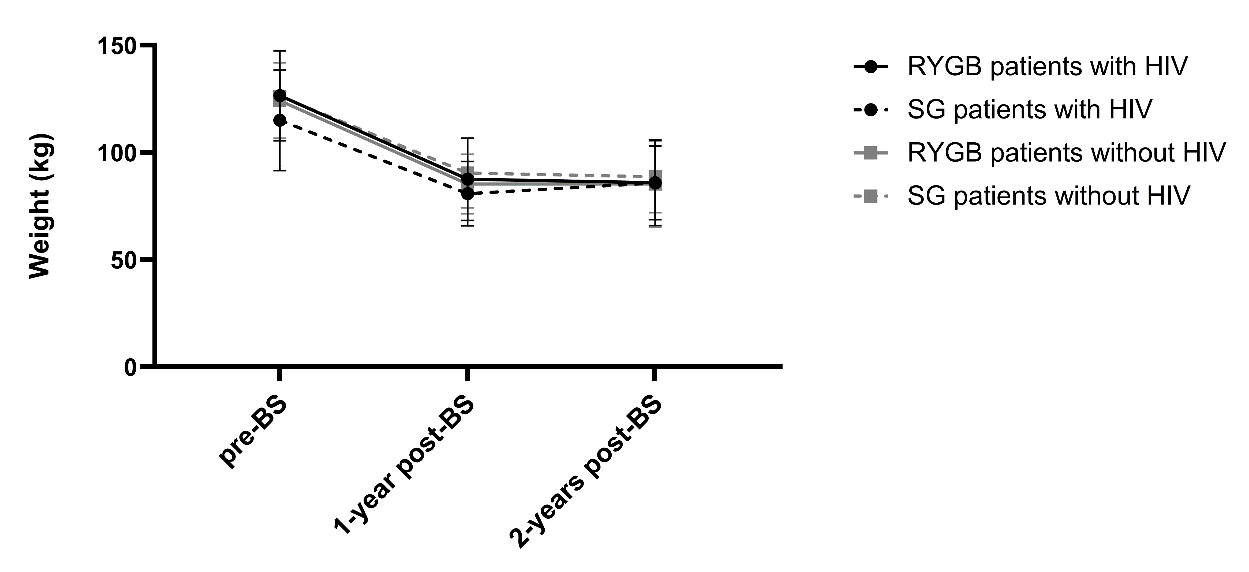


Figure S2 Mean weight in kg (SD) of people with and without HIV up to 2-years after bariatric surgery (BS) stratified by surgery type

Table S3. Characteristics of the surgical complications up to -1-year post-BS

|  | **People living**  **With HIV  (N = 27)** | **Uninfected controls**  **(N = 168)** |
| --- | --- | --- |
| **Patients with complications (N (%))** | 0 (0.0) | 21 (12.5) |
| **Complication period (N (%))** | | |
| Short term (<30 days) without readmission | N/A | 3 (1.8) |
| Short term (<30 days) with readmission | N/A | 8 (4.8) |
| Long term (>30 days) without readmission | N/A | 2 (1.2) |
| Long term (>30 days) with readmission | N/A | 8 (4.8) |
| **Clavien Dindo Classification (N (%))** | | |
| Degree I | N/A | 2 (1.2) |
| Degree II | N/A | 4 (2.4) |
| Degree III | N/A | 13 (7.7) |
| Degree IV | N/A | 0 (0.0) |
| Degree V | N/A | 0 (0.0) |
| Unknown | N/A | 2 (1.2) |
| **ICU-admission (N (%))** | N/A | 0 (0.0) |
| **Type of complication (N (%))** | | |
| Bleeding | N/A | 1 (0.6) |
| Needle leakage | N/A | 3 (1.8) |
| Anatomic stricture | N/A | 1 (0.6) |
| Wound infection | N/A | 1 (0.6) |
| Vomiting | N/A | 1 (0.6) |
| Metabolic complications | N/A | 1 (0.6) |
| Deficiency | N/A | 1 (0.6) |
| Electrolyte disturbances | N/A | 1 (0.6) |
| Unknown | N/A | 11 (6.5) |

**References:**

1. Brethauer, S.A., et al., Standardized outcomes reporting in metabolic and bariatric surgery. Surg Obes Relat Dis, 2015 May-Jun. 11(3): p. 489-506.
